# Late diagnosis of coarctation of the aorta in a 44-year-old male: a case report

**DOI:** 10.1186/s12872-020-01753-1

**Published:** 2020-11-03

**Authors:** Weijian Luo, Jilin Li, Xiaojun Huang, Xiangna Cai

**Affiliations:** 1 Department of Cardiology, Second Affiliated Hospital of Shantou University Medical College, No. 69 Dongxiabei Road, Jinping District, Shantou, 515041 Guangdong Province People’s Republic of China; 2 Department of Plastic Surgery, First Affiliated Hospital of Shantou University Medical College, Shantou, 515041 Guangdong China

**Keywords:** Case report, Hypertension, Renal inadequacy, Heart failure, Balloon angioplasty, Stent placement, Coarctation of the aorta

## Abstract

**Background:**

Coarctation of the aorta is a rare congenital disease. In adults, the main manifestations include hypertension, weak or absent femoral pulses, heart failure, and left ventricular hypertrophy.

**Case presentation:**

We present a case involving a late diagnosis of coarctation of the aorta detected during aortography in a 44-year-old man. The patient underwent stent implantation and aortoplasty. After 2 years of follow-up, the patient was in good condition.

**Conclusions:**

This case shows that coarctation of the aorta can be cured and that hypertension caused by the condition can be controlled to some extent with medication. Based on our findings, we recommend a detailed physical examination for all patients suspected of having coarctation of the aorta; the examination should include blood pressure measurements of both the upper and lower extremities. The case of coarctation of the aorta is not common or easy to be found in medium-aged population. Better BP control, earlier repair, and transcatheter intervention may result in a good outcome in that case.

## Background

Coarctation of the aorta is a rare congenital heart disease characterized by aortic narrowing. Though it is usually detected during childhood, some patients may remain asymptomatic until adulthood [1–4]. Adults usually present with hypertension, weak or absent femoral pulses, heart failure, and left ventricular hypertrophy. In the hopes of bettering diagnosis and treatment for coarctation of the aorta, we herein present a case involving late diagnosis of the condition.

## Case presentation

**Fig. 1:**
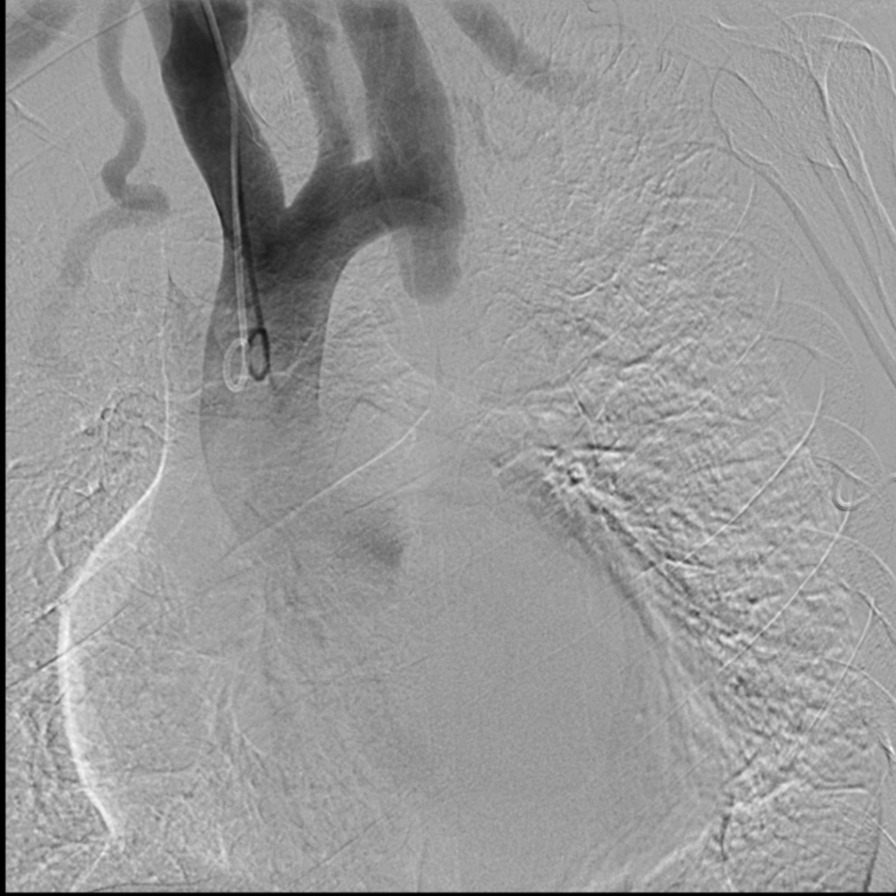
Angiogram demonstrating aortic coarctation (the proximal ascending aorta was slightly narrowed and the proximal descending aorta was severed)

**Fig. 2:**
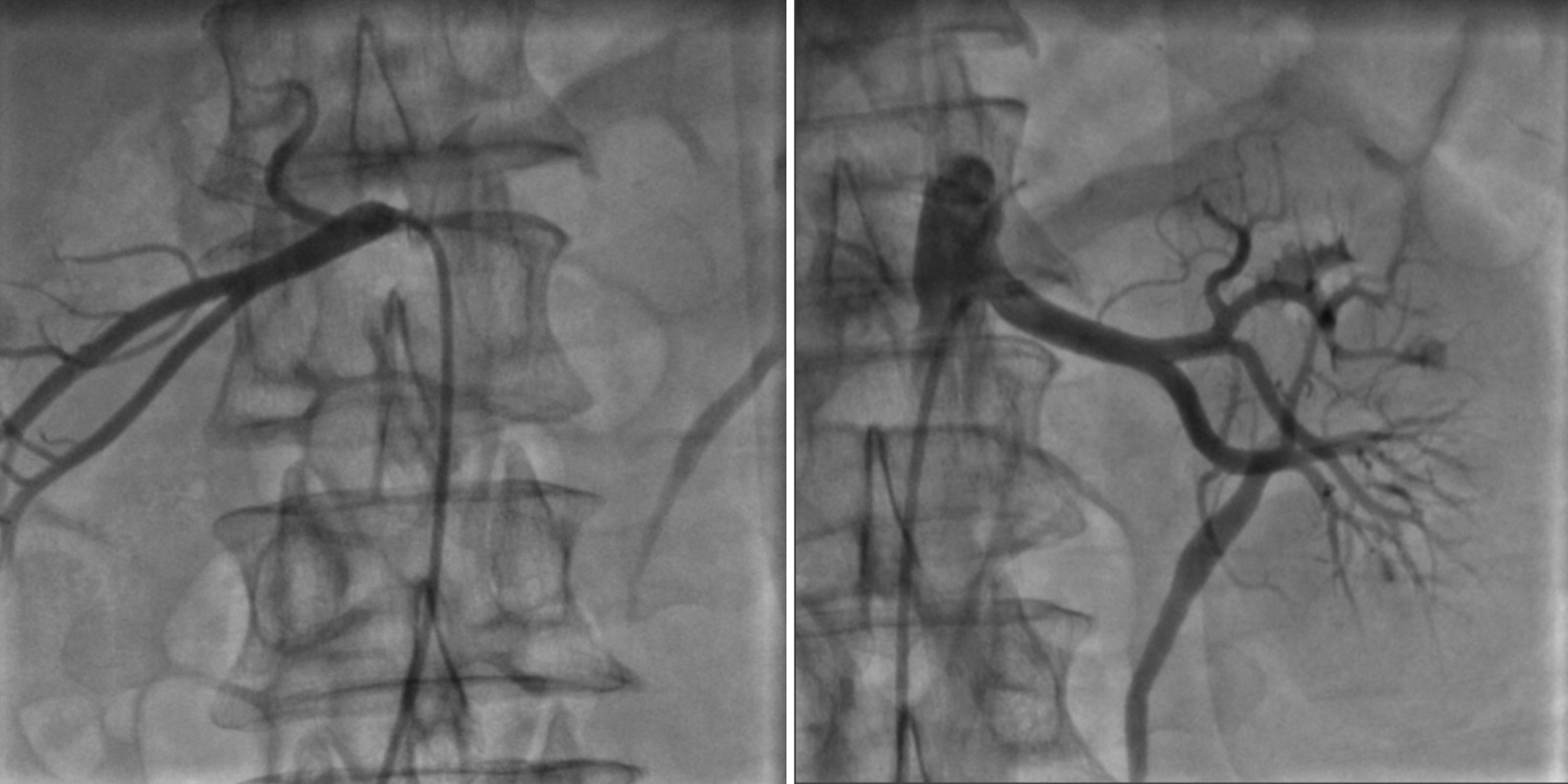
Renal angiogram demonstrating no significant obstruction

A 44-year-old man was hospitalized for 6 days in June, 2012 because of hypertension (> 4 years) (Table 1). During hospitalization, his blood pressure was controlled at around 135/66 mmHg. The cause of hypertension could not be detected despite a thorough laboratory examination and transthoracic echocardiography (TTE). His blood pressure was poorly controlled with benazepril and metoprolol after leaving the hospital. He did not undergo follow-up after discharge. He was hospitalized again for 21 days in February, 2016 due to chest tightness (Table 1). A physical examination showed his blood pressure was 200/105 mmHg. His blood pressure in the lower limbs and femoral pulses were not determined. Results from laboratory tests showed an NT-proBNP level of 24,548 pg/mL and a creatinine level of 152 µmol/L. Echocardiography revealed cardiomegaly, moderate pulmonary hypertension, decreased left ventricular systolic function, and an ejection fraction (EF) of 34% (Table 2). Given the patient had hypertension with high levels of NT-proBNP, the initial differential diagnosis focused on cardiac ischemia and hypertensive cardiopathy. Due to the possibility of cardiac ischemia, the patient underwent angiography (Table 1). Coronary angiography revealed 50% stenosis in the middle segment and proximal end of the left anterior descending artery, 70% stenosis in the distal end of the left circumflex artery, 80% stenosis in the middle segment of the first septal branch, and 95% stenosis in the middle segment of the third septal branch. We implanted two stents in the third septal branch. The patient then underwent aortography and renal angiography. Aortography showed the proximal ascending aorta was slightly narrowed, the proximal descending aorta was severely stenosed, and the mid-distal segment was supplied by collateral circulation (Fig. 1). Renal angiography results revealed no significant obstruction (Fig. 2). Due to the lack of adequate preoperative preparation, the coarctated segment was not treated. After discharge, regular treatment with amlodipine besylate, Metoprolol, spironolactone, furosemide and α-ketoacid was initiated. His blood pressure was controlled around 140/60 mmHg.

**Table 1:** A table provides the timeline of this case

Timeline			
Date	Summaries from initial and follow-up visits	Diagnostic testing (including dates)	Interventions
Iune, 2012 for 6 days	Hospitalized for hypertension		Benazepril and metoprolol
February, 2016 for 23 days	Hospitalized again owing to chest tightness	NT-proBNP, 24548 pg/mL. Creatinine, 152 μmol/L Echocardiography: EF (%), 34	
February, 2016		Angiography	
March, 2016 to April, 2016 for 25 days	Hospitalized again owing to chest tightness	Creatinine, 223 μmol/L	
April, 2016			Stent implantation and aortoplasty
Sept, 2018	Be in good physical condition	Creatinine, 149 μmol/L. Echocardiography:EF (%), 34

**Table 2:** Transthoracic echocardiography

	EF (%)	LVDd (mm)	LVsd (mm)	RA (mm)	RV (mm)
2016-2-3	34	63	52	65*47	29
2018-9-26	56	67	53	43*30	18
Change	64.7%

**Fig. 3:**
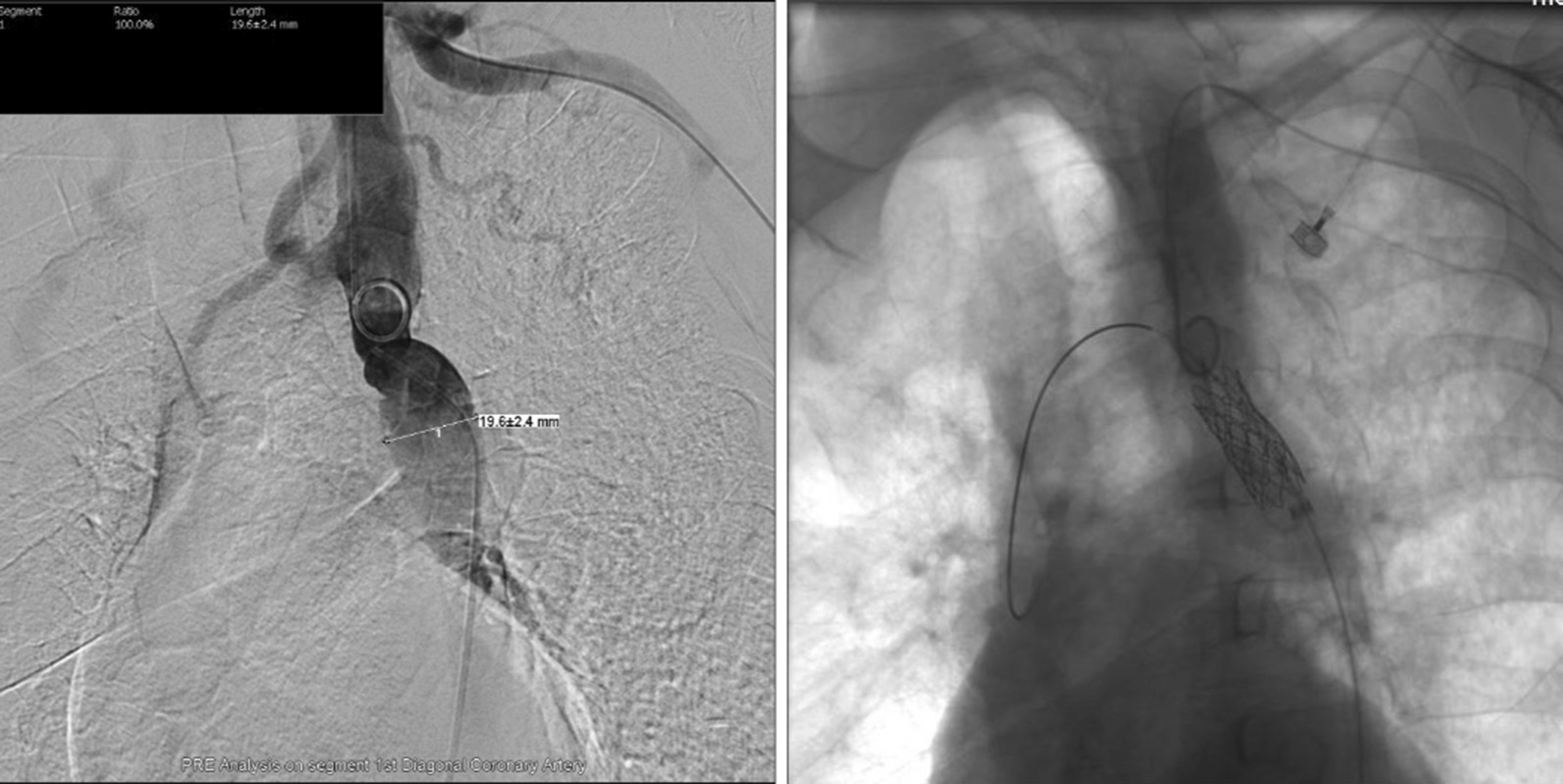
Angiogram demonstrating balloon sizing of coarctation and stent deployment (a stent was implanted in the proximal descending aorta)

The patient was again hospitalized for 25 days from March, 2016 to April, 2016 due to chest tightness (Table 1). Laboratory test results showed that his creatinine levels had increased to 223 µmol/L (Table 3). There was no significant obstruction in the renal artery; therefore, we considered the renal inadequacy was caused by insufficient perfusion. Based on the aortography results, we performed stent implantation and aortoplasty (Table 1).The patient first underwent balloon angioplasty, then stent implantation (CVRDCP8Z39, NUMED).Stent implantation and deployment were performed successfully (Fig. 3). After stent implantation, the patiAnt’s blood pressure and serum creatinine level decreased significantly. At the 2-year follow-up visit, the patient was found to be in good physical condition (Table 1). His blood pressure was controlled at around 130/50 mmHg with valsartan amlodipine and indapamide. Echocardiography revealed an EF of 56% (Table 2), and laboratory results showed a creatinine level of 149 µmol/L (Table 3).

**Table 3:** Creatinine levels

	Cr (umol/L)	CCr (ml/min)
2016-4-3 (before stent implantation)	223	31.91
2016-4-7 (after stent implantation)	164	43.39
2017-6-2 (after 1 year follow-up)	149	47.76
Change	74	15.85

## Discussion and conclusions

Coarctation of the aorta is a rare congenital disease which accounts for only 5–8% of all congenital heart defects, and the prevalence of isolated forms is about 3 per 10,000 live births [1]. The natural course of patients with coarctation of the aorta may be complicated by left-sided heart failure, intracranial hemorrhage, infective endocarditis, aortic rupture/dissection, premature coronary and cerebral artery disease, and associated heart defects [1]. With recent developments, coarctation of the aorta can be diagnosed and repaired early, which has changed the nature of the disease’s progression. It is still unknow the prevalence of aortic coarctation in population especially in hypertension.

Though most cases involving coarctation of the aorta are detected in children, some patients are diagnosed and undergo repair procedures during adulthood [2–4]. In this case, a 44-year-old man was hospitalized for hypertension, heart failure, and renal insufficiency, which made his condition more complicated than those of other patients with coarctation of the aorta. In adults, diagnosis of coarctation of the aorta is based on medical history, physical examination, and imaging studies. It is very important of physical examination and imaging studies for adult hypertension with cardiomegaly and heart failure and renal insufficiency. In addition to hypertension and chest tightness, adults with coarctation of the aorta can present with other symptoms such as arrhythmia and heart failure aortic dissection [5]. Many imaging modalities can be used to detect coarctation of the aorta, with catheter angiography being the golden standard for diagnosis [5]. In this case, the patient underwent TTE and catheter angiography, and the diagnosis was confirmed based on the angiography findings. CTA is an important imaging technique for evaluating coarctation and collateral circulation. However, the patient was not referred for CTA because of renal insufficiency. We did not determine the patient’s blood pressure in the lower limbs or his femoral pulses; retrospectively, we may have found that the blood pressure of the lower extremities did not match that of the upper extremities. At first, as this crucial information was not collected, we ignored the possibility of coarctation of the aorta. With amlodipine besylate, metoprolol, spironolactone, furosemide, and α-ketoacid, his blood pressure was controlled and his renal function improved to some extent before operation. Indications for the treatment of coarctation of the aorta include systemic hypertension, > 50% luminal narrowing, or both [6].

Without treatment, the prognosis for patients with coarctation of the aorta is poor.According to Campbell’s report,most of them died for cardiovascular event before 50 years old [7]. Guidelines regarding indications for intervention exist for both children and adults with coarctation, which include a peak­to­peak gradient ≥ 20 mmHg or lesser gradients when there is significant anatomic evidence of narrowing on imaging with extensive collateral flow [8, 9]. Other factors that may be considered include the presence of systemic hypertension, additional cardiac defects and/or single ventricle physiology, left ventricular hypertrophy, or elevated left ventricular end diastolic pressure [8–11].

At present, patients with coarctation can be cured by several surgical techniques [12]. In contrast to native coarctation, balloon angioplasty is often the preferred intervention for recurrent coarctation in children [9]. An Observational Study by the Congenital Cardiovascular Interventional Study Consortium showed that stent patients had lower acute complications compared with surgery patients, although they were more likely to require a planned reintervention [13]. Considering of the patient’s anatomy and age and the safety of treatment, we eventually chose transcatheter procedure to relief the coarctation. Accurate diagnosis and management of this severe case already stopped the deterioration of heart and renal failure. This case showed that hypertension caused by coarctation of the aorta can be controlled to some extent with drugs and that the condition can be cured, even if the aorta is severely stenosed. Based on this case, we suggest detailed physical examination for all patients with hypertension and cardiomegaly. The careful blood pressure measurement should include both the upper and lower extremities. We also suggest that coarctation of the aorta be included in the differential diagnosis of hypertension. In conclusion, better BP control, earlier repair, and risk factor modification may have a good outcome without early or aggressive CVD.As for patient follow-up, patients with coarctation of the aorta must be followed by a cardiologist throughout their lifetime and imaging of the repaired coarctation should be performed regularly.

## Data Availability

All data associated with this study can be found in the paper or the Supplementary Materials.

## Abbreviations

EF: Ejection fraction
TTE: Transthoracic echocardiography
CTA: Computed tomography angiography
LVDd: Left ventricular diastolic diameter
LVsd: Left ventricular systolic diameter
RA: Right atrium
RV: Right ventricle
Cr: Creatinine
CCr: Creatinine clearance
